# Evaluating the Suitability of the Plantaris Tendon for Sports Trauma Reconstruction and a Predictive Model of Tendon Length Based on Height and Leg Length

**DOI:** 10.3390/jcm12216932

**Published:** 2023-11-05

**Authors:** JeongHyun Park, Kwang-Rak Park, Hyung-Wook Kwon, Yu-Jin Choi, Mijeong Lee, Digud Kim, Sung Wook Choi, Jaeho Cho

**Affiliations:** 1 Department of Anatomy & Cell Biology, School of Medicine, Kangwon National University, Chuncheon 24341, Republic of Korea; jhpark@kangwon.ac.kr (J.P.); kwenhw@naver.com (H.-W.K.); police5565@hanmail.net (Y.-J.C.); toff337@hanmail.net (M.L.); oe5235@naver.com (D.K.); 2 Department of Anatomy, College of Korean Medicine, Sangji University, Wonju 26339, Republic of Korea; airboba@naver.com; 3 Division of Forensic Medical Examination, National Forensic Service, 10, Ipchun-ro, Wonju 26460, Republic of Korea; chsw827@korea.kr; 4 Department of Orthopaedic Surgery, Chuncheon Sacred Heart Hospital, Hallym University, 77, Sakju-ro, Chuncheon 24253, Republic of Korea

**Keywords:** cadaver, sports trauma, anatomic reconstruction, plantaris tendon, tendon graft, tendon length, linear regression

## Abstract

This study evaluates the suitability of the plantaris tendon (PT) as a tendon graft donor for sports trauma reconstruction and proposes a predictive model for estimating PT length by using an individual’s height and leg length. Anatomical dissection of 50 cadavers (32 males and 18 females) yielded precise measurements of PT length and width while also recording height and leg length. Among the lower limbs, 89% were suitable for at least one recommended graft suitability criterion. In addition, PT length exhibited robust positive correlations with height and leg length. Predictive equations were established for estimating the PT length based on leg length and height with consistency across sexes and sides: PT length = 0.605 + 0.396 × leg length (r = 0.721) and PT length = 1.480 + 0.193 × height (r = 0.626). This study underscores the grafting potential of the PT, providing a predictive tool that can aid surgeons in addressing tendon graft challenges within sports trauma scenarios.

## 1. Introduction

The plantaris muscle (PM) is a fusiform muscle with a short belly and a long, thin tendon [1,2]. The plantaris tendon (PT) is nicknamed the “freshman’s nerve” because students new to dissecting cadavers often confuse the thin, rope-like tendons with nerves [3]. PM originates from the deep side of the lateral head of the gastrocnemius muscle and extends to the belly, then turns into a tendon while running distally. The PT descends across the space between the soleus and gastrocnemius and runs distally from the medial border of the Achilles tendon. Its final destination is the calcaneus [1,2,4]. The PT is the longest of the tendons in the human body and is three to four times longer than the plantaris muscle belly (PB).

In a previous paper that meta-analyzed the PM, it was reported that the percentage of PM that is absent varies by race, with a mean value of about 7%. Additionally, in a previous literature review targeting the Korean population, it was reported that the PT did not exist in 6.5% of cases. PTs can be classified into several types depending on the insertion type, with the location and distribution area of PT insertion into the calcaneus as the criteria. This has been examined not only in the Korean population but also in various races, which are generally classified into six types. However, there have been no reports on whether the type of PT insertion affects the length of the PT, and further research on the correlation between the type and length may be included in the review criteria for eligibility as a tendon graft donor [2,5,6].

To date, the PT has been used for tendon grafts in the context of sports trauma reconstruction, including lateral ankle ligament reconstruction, rotator cuff tear reconstruction, and anterior cruciate ligament reconstruction [7,8,9]. In addition, the possibility of using it as a donor that can replace the tendinous cords or papillary muscle has been suggested [10]. Several more advanced methods of tendon grafting are being developed to enhance the strength of reconstruction through the use of additional suturing of the PT in Achilles tendon reconstruction surgery [11,12].

A review of the literature on the PT shows that tendon grafts provide sufficient lateral elasticity as donors, with high tensile strength and stiffness values compared to those of other grafts. Therefore, the PT could be a viable candidate as a donor for tendon grafts [13,14]. Harvey et al. [13] proposed minimum suitable width and length values of the PT for tendon grafts of 1.5 mm and 15 cm, respectively, with maximum values of 3.0 mm and 30 cm, respectively.

The use of autografts for tendon reconstruction has several potential advantages. Tendon harvesting is easy and can minimize inflammatory reactions, improving healing. Additionally, autografts have potential biological advantages over allograft materials in tendon reconstruction [7]. Although the PT has been recognized for its usefulness as a tendon graft donor, few studies have been conducted to establish or predict the possibility of the PT satisfying length requirements as such a donor.

The primary objective of this study is to assess the suitability of the length and width of the PT for use as a graft donor in sports trauma reconstruction while specifically focusing on the Korean population. We also propose a predictive model that employs an individual’s height and leg length to estimate the length of the PT to facilitate its utilization as a graft donor in diverse clinical scenarios.

## 2. Materials and Methods

A total of 50 cadavers (32 males and 18 females) embalmed with a 10% formalin mixture were included in the study. All cadavers were between 44 and 97 years old (76.04 ± 11.22 years), and there were 64 male limbs and 36 female limbs out of the total of 100 lower limbs (Table 1). Lower limbs with evidence of malformation or surgical intervention in the dissected area were excluded. All cadavers were donated through an accredited body donation program for education and research. We received approval from the Ethics Committee of our institution for this study (Institutional Review Board number: CHUNCHEON NON-2021-001).

The legs were dissected according to normal anatomical procedures. The body was placed in a prone position, and a support was used for the ankles for careful dissection. After confirming the origin of the gastrocnemius in both condyles of the femur, it was removed. The origin of the PM was found near the lateral epicondyle, slightly above the lateral head of the gastrocnemius muscle. The PM was then sequentially dissected, starting from the proximal belly to the insertion of the distal tendon. Finally, the entire PM was exposed.

**Figure 1:**
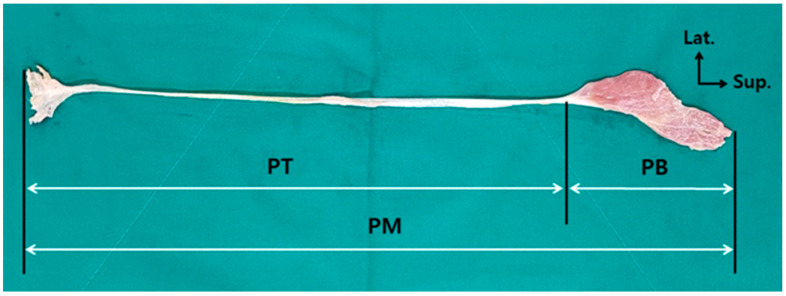
Measurement of the length of the plantaris muscle. PM, plantaris muscle; PT, plantaris tendon; PB, plantaris muscle belly.

The height was measured in a supine position; the chin was pulled toward the body, the knees were stretched as far as possible, and the ankles were placed in a neutral position. Then, the distance from the top of the head to the tips of the heels was measured. The leg length was measured by using the method of measuring the distance from the anterior superior iliac spine to the tip of the medial malleolus, which was suggested by Gogia and Braatz [15], in the same position as that used for the height measurement. The length of the PM was measured by dividing it into the PB and the PT, and the total length was calculated by adding the two length values. The tendon width was measured at the midpoint of the PT. All measurements related to the PM were taken, with the PM remaining intact at its origin and insertion (Figure 1). All lengths were independently measured once each by two researchers. The height and leg length were measured by using a general tape measure (Tajima Tool Corporation, Torrance, CA, USA), and the lengths and widths of the PB and PT were measured with electronic digital calipers (Sincon Corporation, Los Angeles, CA, USA). The unit of measurement for all lengths, including the height and leg length, was 0.1 cm, and the PT width was measured in units of 0.1 mm.

### Statistical Analysis

All statistical analyses were performed by using IBM SPSS Statistics version 23.0 (IBM Co., Armonk, NY, USA). The inter-class correlation coefficient (ICC) was used to evaluate the inter-class reliability for all measurements. All length and width variables were assessed with a normality test. A comparison of the means of all length and width variables according to sex was analyzed by using an independent sample *t*-test. Ratios involving the height, leg length, and PT length were calculated as the mean (standard deviation) of the length variables. Correlation analysis was performed to identify the correlations between the height, leg length, and age and the PT length. The higher the correlation coefficient (Pearson r), the stronger the correlation between variables. Multiple regression analysis was performed to evaluate the factors that were significant in the PT length variables for sex and leg length. Linear regression analysis was performed to derive a regression equation that could predict PT length by using leg length. All results were considered statistically significant when the *p*-value was less than 0.05.

## 3. Results

As a result of generating inter-class correlation coefficients for all measurements, Cronbach’s alpha was 0.96 in length and 0.97 in width, showing the excellent reliability (over 0.81) of all results [16]. The mean height was 167.17 ± 5.08 cm for males and 152.75 ± 5.79 cm for females, and the leg length was 83.52 ± 3.61 cm for males and 76.79 ± 3.88 cm for females. The overall mean height was 161.98 ± 8.76, and the leg length was 81.10 ± 4.91. The mean PM length was 43.12 ± 3.06 cm for males and 38.66 ± 2.28 cm for females, the PB length was 9.25 ± 1.75 cm for males and 7.94 ± 0.99 cm for females, and the PT length was 33.86 ± 2.37 cm for males and 30.71 ± 2.00 cm for females. The overall mean of the PM length was 41.51 ± 3.52, that of the PB length was 8.78 ± 1.64, and that of the PT length was 32.73 ± 2.70. The mean width of the PT was 2.92 ± 0.90 mm for males and 3.01 ± 0.85 mm for females. The overall mean of the PT width was 2.95 ± 0.88. When compared according to sex, all length variables were significantly longer in males than in females, but there was no difference in width. There were no significant differences in PM length and width between the right and left sides (Table 2).

**Figure 2:**
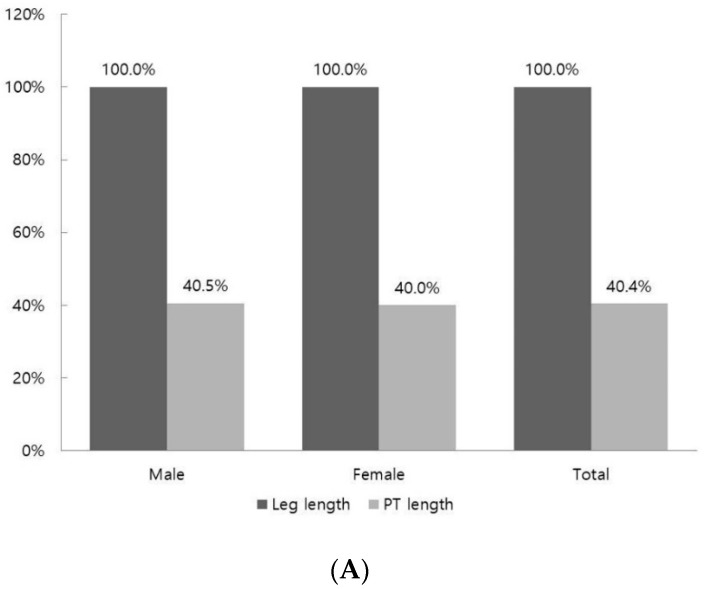

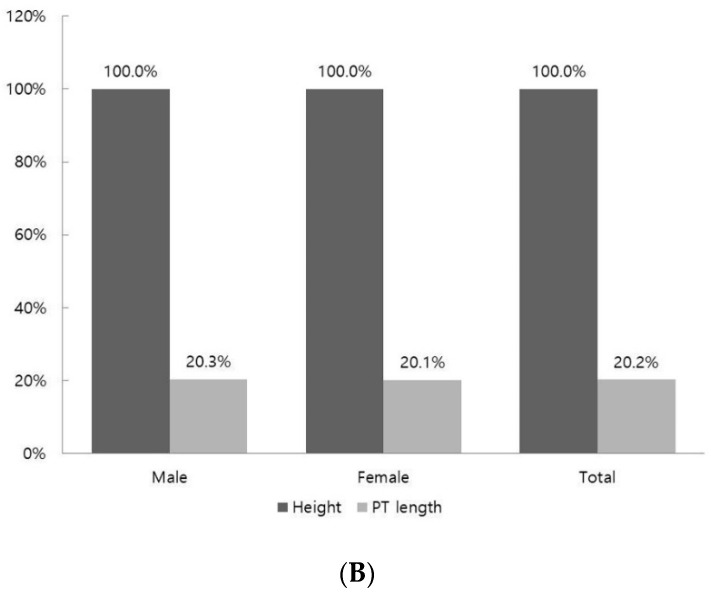
Ratios of the plantaris tendon based on the leg length and height. (**A**) PT length ratio based on leg length; (**B**) PT length ratio based on height; leg length, straight distance from the anterior superior iliac spine to the tip of the medial malleolus; PT, plantaris tendon.

The ratio of the PT length calculated based on 100% of the leg length was 40.4%. There was no difference in the ratio according to sex, with 40.5% for males and 40.0% for females (Figure 2A). The ratio of the PT length calculated based on 100% of the height was 20.2%. There was no difference in the ratio according to sex, with 20.3% for males and 20.2% for females (Figure 2B).

There was no statistically significant correlation between PT length and age (r = −0.083, *p* = 0.412). However, there was a positive correlation between PT length and height (r = 0.626, *p* < 0.001), and a positive correlation was also found between PT length and leg length (r = 0.721, *p* < 0.001). There was no statistically significant correlation between PT length and width (r = −0.047, *p* = 0.643) (Table 3).

In the multiple regression analysis, PT length had a significant relationship with both leg length and height (*p* < 0.001). The adjusted R^2^ value for the leg length and tendon length was 0.523, and the adjusted R^2^ value for height was 0.403. However, there was no significant relationship between the PT length and sex (*p* = 0.101) (Table 4).

**Figure 3:**
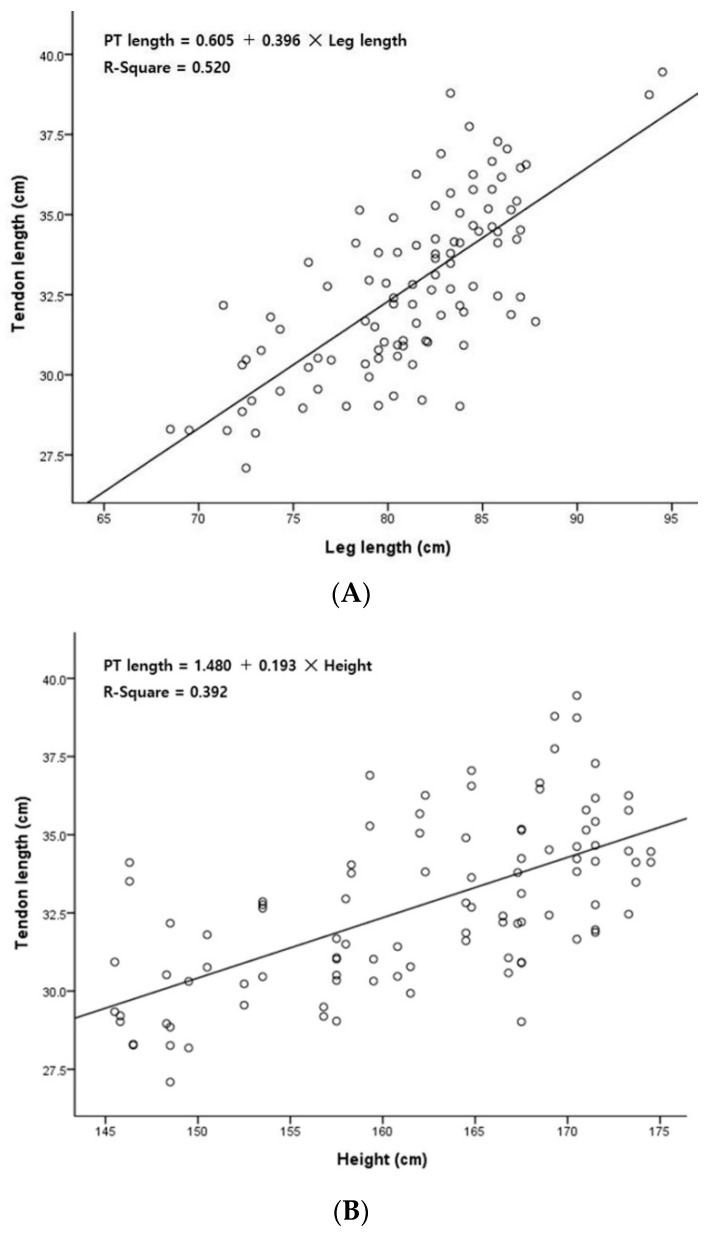
Scatterplots of the length variables. (**A**) Scatterplot of leg length versus plantaris tendon length; (**B**) scatterplot of height versus plantaris tendon length; PT, plantaris tendon.

As a result of a simple linear regression analysis between PT length and leg length, R^2^ = 0.520, which had an explanatory power of 52.0% and showed that there was a significant relationship (*p* < 0.001). An equation for predicting the PT length (x, cm) based on the leg length (y, cm) variable was derived: PT length = 0.605 + 0.396 × leg length (Table 5) (Figure 3A).

As a result of the simple linear regression analysis between PT length and height, R^2^ = 0.392; thus, this relationship had an explanatory power of 39.2% and was a statistically significant relationship (*p* < 0.001). An equation for predicting the PT length (x, cm) based on the height (y, cm) variable was derived: PT length = 1.480 + 0.193 × height (Table 5) (Figure 3B).

The two recommended criteria for determining tendon graft suitability proposed by Harvey et al. [13] were applied to the results of this study, which targeted Koreans. There were 80 (80%) cases in which the tendon length was more than 30 cm and the tendon width was more than 15 mm. There were 44 (44%) cases in which the tendon length was more than 15 cm and the tendon width was more than 3.0 mm. As a result, 89 (89%) of the lower limbs met at least one of the recommended criteria (Table 6).

## 4. Discussion

In this study, we presented a regression model for predicting PT length in the Korean population, and 89% of the patients obtained positive results in the analysis of their suitability as tendon transplant providers. The PT is widely used as a donor when reconstructing damaged ankle ligaments or as a reinforcement in reconstructive surgery for Achilles tendon rupture [9,11,12]. Therefore, the results of this study not only showed that the PT can have a high frequency of use as a donor for tendon transplantation, but its length can also be relatively simply predicted based on height or leg length, making this a clinically applicable model.

In this study, the mean length of the PT was 32.7 ± 2.7 cm. In a previous study, the PT length of Indians was similar to that found in our study, but the PT length of Iranians was shorter at 23.3 ± 6.8 cm [17,18]. The length of the PT was found to be different for each race. The PT length in males was longer than that in females (*p* = 0.000) (Table 2). However, as a result of comparing the ratio of the PT length to leg length according to sex, males were found to have a value of 40.5%, and females were found to have a value of 40.0%; the ratio of PT length to height was 20.3% for males and 20.1% for females (Figure 2). In addition, there was no significant relationship between PT length and sex in the multiple regression analysis (*p* = 0.101) (Table 4). There was no difference between left and right in terms of PT length. Nayak et al. [17] reported that there was no difference in length between the left and right sides when comparing the difference between sides in terms of PT length in Indians, and they showed results similar to those of this study. Considering the above results for PT length, when using the PT as a tendon graft donor, the sex and side may not be considered, so this can be usefully applied clinically.

The mean PT width in this study was 2.9 ± 0.8 mm. In a previous study, while some population groups had similar widths to those in our results [18], ours were wider than those of North Americans by 2.0 ± 0.7 mm [19] and narrower than those of Chinese people by 4.5 ± 1.1 mm [20]. Like the length, the PT width also varied according to the population group. There were no significant differences in PT width in terms of sex or side. Nayak et al. [21] reported that there was no significant difference in width between the left and right in a comparison of the mean PT width on each side, which was similar to our results. Harvey et al. [13] reported that the width of the tendon is the most important factor in determining the suitability of the PT as a tendon graft donor. This is probably why not only the PT length but also the PT width can be used as important factors in determining suitability as a tendon graft donor. According to the results of this study, it seems that the PT width can be applied without considering the sex or side when determining its suitability as a tendon graft donor.

In this study, as a result of calculating the ratio of the PT length to the leg length and height, the ratio of the PT length to the leg length was 40.4%, and the ratio of the PT length to the height was 20.2%. The results of previous studies that calculated the ratio of the fibula length to the PT length were confirmed, but those of previous studies that calculated the ratio of the PT length based on the leg length and height could not be confirmed [22]. However, predicting the ratio of the PT length based on leg length and height is not invasive and can be performed simply on the surface. Therefore, it is thought that the PT length can be used as auxiliary data for judging the PT’s suitability as a tendon graft donor.

Harvey et al. [13] suggested length and width criteria for the suitability of the PT for tendon grafts, and the results of this study were applied to them. PT lengths greater than 30 cm and widths greater than 1.5 mm were found in 80 (80%) of the lower limbs, and PT lengths greater than 15 cm and widths greater than 3.0 mm corresponded to 44 (44%) of the lower limbs. When assessing the frequency of meeting one or both of the conditions, 89 lower limbs were found to be appropriate, and this result indicated that 89% of the measured PTs were suitable donors for tendon grafts. Jakubietz et al. [23] reported that 48 out of 80 lower limbs met the recommendations when measuring the length and width of the PT. These results indicated that 66.7% of grafts with this tendon are suitable. There was a difference in the frequency of suitability between this study on the Korean population and a study on Europeans. In our study of the Korean population, there was a higher ratio (89%) of suitable donors of tendon grafts. The results of this study targeting the Korean population suggest that the PT has high suitability as a donor for tendon grafts in this group in comparison with other population groups. Most previous studies focused only on the presence of the PM and did not evaluate the applicability of tendon grafts. Since there is a possibility that different races may have different levels of suitability, follow-up studies on various races will be needed (Table 6).

In this study, measurements were performed on cadavers that were donated to medical schools for educational and research purposes. For this reason, a relatively large proportion of the subjects were elderly. According to data from Statistics Korea, as of 2021, the average height decreases as age increases, regardless of gender. As a result of this study, the male height was 176 cm, which was similar to Statistics Korea’s statistical results for people aged 60 to 65. The female height of 153 cm was similar to Statistics Korea’s statistical results for people aged 70 to 74. This cannot rule out the possibility that differences in height across age groups also cause differences in PT length. Therefore, to more accurately predict the PT length based on height and leg length, a more extensive and large-scale age-specific survey targeting Koreans should be conducted [24].

This study conducted correlation and linear regression analyses to assess the relationship between PT length and two variables: height and leg length. The results indicated a strong positive correlation between PT length and both height (r = 0.626, *p* < 0.001) and leg length (r = 0.721, *p* < 0.001). Furthermore, the linear regression analysis showed that height explained 39.2% of the variance in PT length (R^2^ = 0.392), while leg length accounted for 52.0% of the variance (R^2^ = 0.520), signifying a moderate level of explanatory power. While these findings suggest substantial relationships, achieving a higher level of explanatory power may necessitate further investigation through follow-up research.

In this study, the correlations between height, leg length, and PT length were analyzed. A positive correlation was established for both height and leg length (*p* = 0.000). Between height and PT length, the Pearson r value was 0.626, and between leg length and PT length, the Pearson r value was 0.721 (Table 3). As a result of the statistical analysis, the influence of sex and age was very weak or absent, so this could not be a constant predictor. Therefore, a simple plastic regression analysis was performed to calculate a prediction equation that could predict the PT length based on the most influential leg length and height, and the results were as follows. The equation for predicting the PT length (x, cm) based on the leg length (y, cm) variable was PT length = 0.605 + 0.396 × leg length. The equation for predicting the PT length (x, cm) based on the height (y, cm) variable was PT length = 1.480 + 0.193 × height. These results are thought to help decide whether to implement a tendon graft by using the PT in clinical practice (Table 5).

There are some limitations to this study. First, since cadavers fixed with formalin were measured, tissue changes may have occurred; thus, the measured values may have been different from the actual values. Second, since there was a small sample size for females in comparison with males, there may be limitations to the accuracy of the comparison between the two groups. Third, the mean age of the corpses was 76.04 ± 11.22 years old, which accounted for a large proportion of elderly subjects, so the mean height was insufficient to explain all ages. Finally, a sample of 50 cadavers (100 lower limbs) may not be sufficient to explain the statistical accuracy, so further studies are needed. However, this study derived an equation for predicting the length of the PT based on leg length for the Korean population. Since this is the first attempt to conduct such a study in the Korean population and is a very rare type of report in foreign countries, continuous and large-scale follow-up studies should be conducted.

## 5. Conclusions

The plantaris tendon shows promise as a graft donor in sports trauma reconstruction. This cadaveric study focused on the Korean population and emphasized the plantaris tendon’s suitability for tendon grafting. Additionally, the predictive model presented here, which utilizes the height and leg length, aids in preoperative planning, thus offering surgeons a valuable and easily accessible asset for managing sport-related injuries.

## Figures and Tables

**Table 1:** Distribution of cadavers by sex and age.

Age	Male	Female	Total
40–49	1	0	1
50–59	3	0	3
60–69	5	2	7
70–79	13	7	20
80–89	9	6	15
90–100	1	3	4
Total	32	18	50

The data are presented as numbers.

**Table 2:** Analysis of differences in length variables according to sex. Leg length, straight distance from the anterior superior iliac spine to the tip of the medial malleolus; PM, plantaris muscle; PB, plantaris muscle belly; PT, plantaris tendon.

Measurement	Male	Female	Total	*p*
Height (cm)	167.17 ± 5.08	152.75 ± 5.79	161.98 ± 8.76	<0.001 ***
Leg length (cm)	83.52 ± 3.61	76.79 ± 3.88	81.10 ± 4.91	
PM length (cm)	43.12 ± 3.06	38.66 ± 2.28	41.51 ± 3.52	
PB length (cm)	9.25 ± 1.75	7.94 ± 0.99	8.78 ± 1.64	
PT length (cm)	33.86 ± 2.37	30.71 ± 2.00	32.73 ± 2.70	
PT width (mm)	2.92 ± 0.90	3.01 ± 0.85	2.95 ± 0.88	0.606

The data are expressed as the mean ± SD; *** *p* < 0.001.

**Table 3:** Correlations of the length of the plantaris tendon with age, leg length, and height.

Pearson Correlation (r)		*p*
Age	−0.083	0.412
Leg length	0.721	<0.001 ***
Height	0.626	<0.001 ***
Width	−0.047	0.643

*** *p* < 0.001.

**Table 4:** Multiple regression analysis of the length variables.

Dependent	Independent	Adjusted *R*^2^	*p*
Tendon length	Leg length	0.523	<0.001 ***
	Sex		0.101
Tendon length	Height	0.403	<0.001 ***
	Sex		0.176

*** *p* < 0.001.

**Table 5:** Linear regression analysis of leg length and height with respect to the length of the plantaris tendon.

	Unstandardized Coefficient		Standardized Coefficient	t	*p*	*R* ^2^
	B	SE	*β*			
(Constant)	0.605	3.126		0.194	0.847	0.520
Leg length	0.396	0.038	0.721	10.296	<0.001 ***	
(Constant)	1.480	3.939		0.376	0.708	0.392
Height	0.193	0.024	0.626	7.944	<0.001 ***	

*** *p* < 0.001.

**Table 6:** Suitability as a tendon graft donor (*n* = 100).

Suitability	Frequency
Tendon length ≥ 30 cm and tendon width ≥ 1.5 mm	80 (80%)
Tendon length ≥ 15 cm and tendon width ≥ 3.0 mm	44 (44%)
More than one suitable	89 (89%)

## Data Availability

The data used to support the findings of this study are available from the corresponding author upon request.
